# Nest characteristics and composition of the colonial nesting Azure-winged magpie *Cyanopica cyanus* in South Korea

**DOI:** 10.7717/peerj.13637

**Published:** 2022-06-29

**Authors:** Ki-Ho Kang, Ki-Baek Nam, Ji-Sub Kim, Jeong-Chil Yoo

**Affiliations:** Korea Institute of Ornithology & Department of Biology, Kyung Hee University, Seoul, Republic of Korea

**Keywords:** Bird nest, Nest characteristics, Nest composition, Cup volume, Azure-winged magpie

## Abstract

Bird nests are crucial for reproductive success since they serve as structures to hold the eggs and nestlings safely. Therefore, the structural characteristics of bird nests have optimally evolved to maximize reproductive success, which are known to be affected by various factors. We gathered information on the nest characteristics such as nest structure and constituent materials in the colonial breeding Azure-winged magpie (*Cyanopica cyanus*) and investigated the relationship between ecologically relevant factors and the size and mass of the nests. The Azure-winged magpie nest can be deconstructed into an outer nest and an inner cup, and the type and mass of materials used for the construction of each part varies. Compared to the inner cup, the outer nest, which constitutes the overall shape of the nest, is composed of relatively harder materials, such as branches and soil. In contrast, the inner cup, which is the part where birds directly incubate eggs and raise nestlings, is composed of more flexible and softer materials, such as fiber and moss. We found that there was no relationship between nest characteristics and ecologically relevant factors. However, as the breeding season progressed, the volume of the inner cup decreased with increasing ambient temperatures. Our results show that Azure-winged magpies use differing materials for structurally distinct parts of the nests during construction. The results also indirectly suggest that the choice regarding the amount of insulating materials relative to changing temperatures during the breeding season may be one of the more significant adaptive strategies in the nest-building behaviors of Azure-winged magpies.

## Introduction

A bird nest provides a safe space for birds to lay eggs and raise nestlings (Heenan, 2013; Møller et al., 2014; Wysocki et al., 2015). Breeding individuals expend significant time and energy to build nests for successful reproduction (Hotta, 1994; Berg et al., 2006). The shape and size of a nest may vary from species to species or even within a species, depending on the nest site, the materials used in its construction, the need for temperature control, and the functional link between the temperature and the humidity of the breeding site. Ultimately, bird nests have optimally evolved to ensure successful reproduction (Wimberger, 1984; Heeb, Kölliker & Richner, 2000; Deeming & Biddle, 2015).

In some bird species, the nest is a structure constructed with various materials, and its constituent parts can be easily distinguished based on these materials (structural materials or lining materials) used (Mainwaring et al., 2014). The outer nest is mainly composed of materials such as branches or soil, and its shape provides a secure space for breeding individuals as well as their eggs and nestlings. Thicker and harder materials are used compared to those used to construct the inner cup (Biddle, Deeming & Goodman, 2015). The inner cup is the inside part of the nest and is typically composed of softer and insulating materials, such as moss, fiber, and feathers, which creates a suitable microclimate for birds to incubate their eggs and keep their nestlings warm (Hansell, 2000, 2005; Windsor, Fegely & Ardia, 2013).

Characteristics of the nest including size, mass, and cup volume are known to be affected by various factors. First, the timing of breeding may affect the size and mass of the nest or the cup. In particular, it has been reported that the amount of insulation materials utilized in nest-building is related to temperature changes in the nest and its surroundings during the breeding season (Mainwaring & Hartley, 2008; Britt & Deeming, 2011). Therefore, the size and mass of the nest or the cup would also vary accordingly. (Deeming et al., 2012; Akresh, Ardia & King, 2017). Second, the nest position from the ground may affect its overall nest size or the volume of the cup. Breeding individuals may choose a different location for nest building within a tree due to predation pressures in relation to predator types (Tabib et al., 2016). For this reason, it is expected to affect the size or mass of the nest in relation to the nest position for its survival. Lastly, a high breeding density can intensify resource competition within the breeding area (Fretwell & Lucas, 1969; Forero et al., 2002; Sillett, Rodenhouse & Holmes, 2004). Accordingly, the degree of competition for nest materials is expected to vary relative to breeding density.

An investigation of the relationship between the aforementioned ecologically relevant factors and nest characteristics can indirectly provide information for studying the nesting behavior patterns of breeding individuals, and it ultimately may contribute to an increased understanding of their nest-building strategies. In this study, first, we investigated the breeding nests of the Azure-winged magpies (*Cyanopica cyanus*) to explore basic information about the structure and materials of their nests. Then, we tested the relationship between ecologically relevant factors and the nest characteristics. Finally, we examined whether the nest characteristics were related to the breeding success in our study population.

## Methods

### Study site and species

The study was conducted in 2018 and 2019 at Joan-myeon, Namyangju-si, Gyeonggi-do, the Republic of Korea (37°33′56.47″N, 127°17′2.87″E). The study area was divided into two sites. Site 1 was a small forest area with various types of trees (Mulberry *Morus alba*, Rosae Multiflorae *Rosa multiflora*, and Japanese yew *Taxus cuspidate*). In contrast, Site 2, was an area of artificially created commercial tree plantations comprised of only a single species of tree (Japanese yew). Both sites are surrounded by agricultural land and farmhouses. Due to the influence of a continental climate, Namyangju-si, the administrative district of the study site, is characterized by high temperatures and humidity in the summer and coldness and dryness in the winter (https://www.weather.go.kr/w/obs-climate/land/past-obs/obs-by-day.do). During the breeding season, the average temperature was approximately 19.9 °C (20.2 °C in 2018; 19.6 °C in 2019), the average precipitation was 133.3 mm (177 mm in 2018; 89.6 mm in 2019), and the average humidity was 62.2% (64.1% in 2018; 60.4% in 2019). The average temperature in the breeding sites increased as the breeding progressed (April 22 to June 28 in the 2018 breeding season, range = 10.2–25.9 °C, *r*^2^ = 0.742, *p* < 0.001; April 22 to July 15 in the 2019 breeding season, range = 9.3–27.9 °C, *r*^2^ = 0.664, *p* < 0.001; Appendix 1).

The Azure-winged magpie, which belongs to the genus *Cyanopica* (Order Passeriformes, Family Corvidae) is a resident bird species that lives in colonies and is widely distributed throughout the Republic of Korea. This species breeds in open-cup nests and has a cooperative breeding system during the breeding season, meaning that helpers may assist in all reproductive stages including nest-building (Valencia, De La Cruz & González, 2003; Oh et al., 2010). According to a previous study (Kim et al., 2015), the Azure-winged magpies in our study area started building nests in late April and fledged nestlings in mid or late June. In addition, they constructed their open-cup nests close to the trunks of trees. Both sexes of breeding individuals are involved in nest building (Valencia, De La Cruz & González, 2003). The average clutch size was seven and there were six nestlings on average during the breeding season.

### Data collection

**Figure 1:**
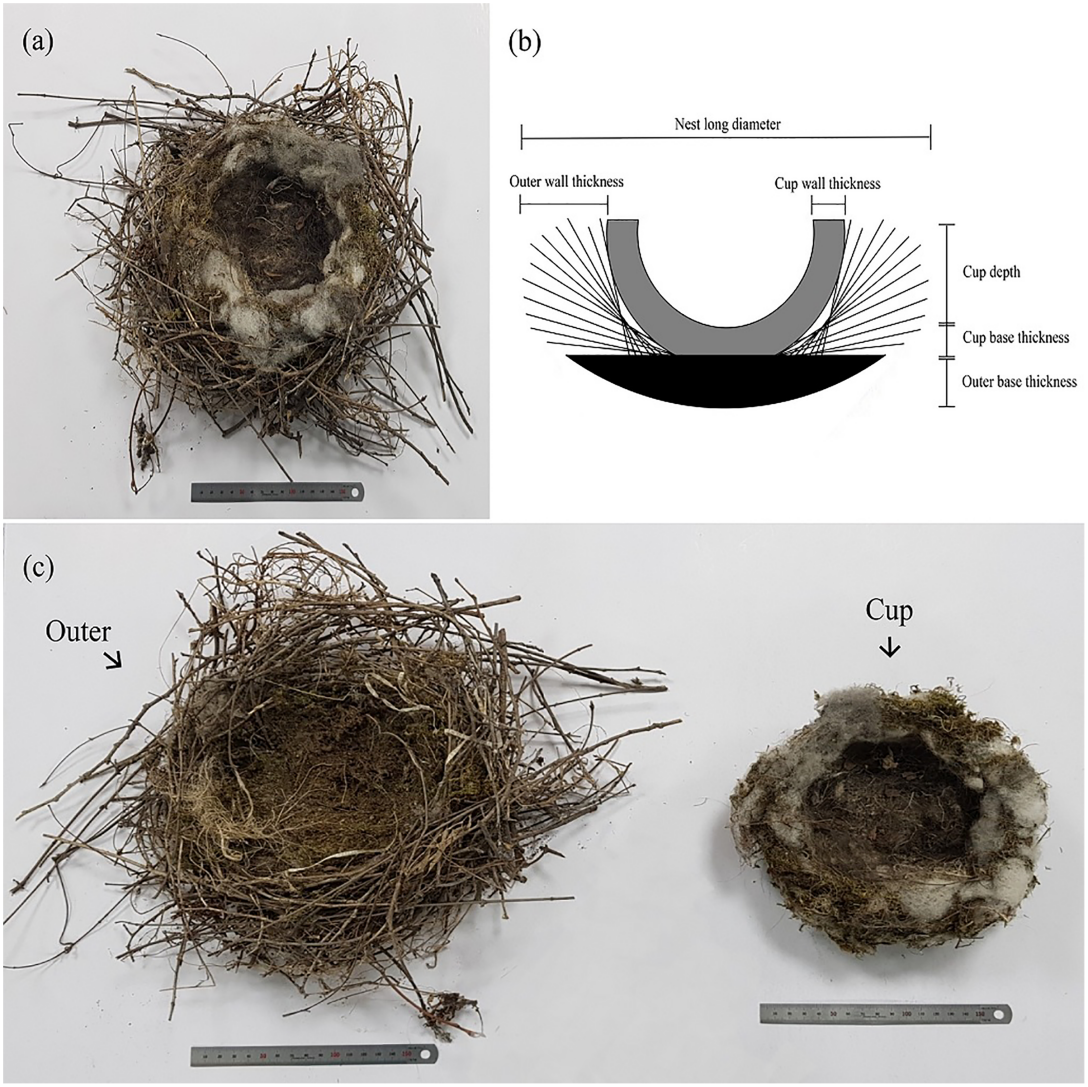
Azure-winged magpie’s nest shape and schematic diagram of the measurements. (A) The actual shape of the nest; (B) schematic diagram of the structure and measurements of the nest; (C) the nest deconstructed into the outer nest and inner cup.

In order to identify the nest locations of Azure-winged magpies, we visited the study sites and observed nest-building behaviors. When we found signs of incipient nest construction in a tree, the location of the nest was recorded on a map. We visited nests every day to check their breeding status (from early April to late July in 2018 and 2019) and to record other ecological information (*e.g*., breeding stage, nest position, number of neighbors). In 2018 and 2019, a total of 28 nests (11 nests in 2018; 17 nests in 2019) were used for observation and collecting information about the nest structure and materials in our analyses. For the deconstruction and measurement of nests (including both breeding successful nests and failed nests), we carefully removed the entire nest from the tree without damage (Fig. 1A). All sample nests were dried under the same condition before the process of dismantling (dried at 40 °C for 72 h).

### Nest deconstruction and measurements

Azure-winged magpies’ nests have clear boundaries between the outer nest and the inner cup. In addition, the materials in each part were different. Therefore, we were able to separate each part with naked eyes (Figs. 1A and 1C). After measuring the overall size and mass of the nest, the outer nest and inner cup were dismantled, taking care not to damage the constituent materials and structural details. Following deconstruction, we measured the size and mass of each outer nest and inner cup respectively. Then, we completely disassembled the outer nest and inner cup to investigate the constituent materials used in the construction of each part.

Measurements of the nest were performed as follows (Fig. 1B). When the uppermost part of the nest was viewed in a plane, the longest length was the nest diameter parallel to the long axis and the shortest length was the nest diameter perpendicular to the long axis. The nest height was measured by the vertical distance from the bottom to the highest part of the nest. Both the cup diameter parallel and perpendicular to the long axis were also measured in the same manner as the whole nest. The cup height was the sum of the cup depth and the thickness of the cup base. The cup depth was measured from the length to the deepest part of the bottom of the cup perpendicular to the cup diameter parallel/perpendicular to the long axis. The cup base thickness was the average of three random measurements made using a thin, pointed tool (*e.g*., needle). The cup wall thickness was also measured in the same way as the cup base thickness. The circumference of the cup was measured by wrapping the upper part of the cup with a flexible string. The cup volume was calculated by measuring the amount of colored sand that filled the cup. The outer base thickness was calculated by subtracting the cup height from the nest height, and the outer wall thickness was calculated by subtracting the cup diameter parallel to the long axis from the nest diameter parallel to the long axis and dividing the value by two.

### Data analysis

First, we presented the structural characteristics of the nest and the amounts of materials used for each part of the nest during two breeding seasons. In order to explore whether the amount of material in an inner cup was affected by the timing of breeding, we tested for the relationship between the amount of moss and fiber (the main materials constituting the inner cup) and timing of breeding. We conducted two analyses using a general linear model, where the moss and fiber (the main materials constituting the inner cup) were treated as a response variable in each analysis, and the timing of breeding was treated as an explanatory variable in all analyses. Second, to investigate the relationship between nest characteristics and ecologically relevant factors, we conducted three analyses using a general linear model, where the size, mass, and cup volume of the nests were treated as a response variable in each analysis (‘nest size’ was defined by calculating the largest surface area of the upper part of the nest) and ecological factors such as year, first-egg date, site, nest position, and number of the neighboring nest were treated as explanatory variables in all analyses. In the case of the cup volume, especially, we used the square root to transform the response variable to facilitate normal and heteroscedastic errors. ‘Timing of breeding’ was defined as the day the first egg was laid in the observation nests. ‘Site’ was divided into two breeding areas depending on the type of environments (site 1 is a forest and site 2 is a plantation). ‘Nest position’ was measured as the vertical distance (m) from the ground level to the bottom of the nest. ‘Number of the neighboring nest’ indicates the number of other active Azure-winged magpie nests within a 10 m radius from the target nest. Finally, to establish whether the breeding success was related to the nest characteristics, we also fitted generalized linear models (GLMs) with a binomial error and a logit link function. The response variable was the breeding success, respectively. For the breeding success, we used a binomial variable set to 0 if the breeding attempts in each breeding stage in the nest were failed and a value of one otherwise. The size, mass of nest and cup volume were treated as explanatory variables. In our models, we used Akaike information criteria (AIC) to refine the model by backwards stepwise deletion, removing terms in the order of increasing χ^2^ value only if dropping them resulted in a model with a lower AIC value. All means are reported as ± standard deviation. All statistical analyses were conducted using the R 3.3.3 program (R Core Team, 2017).

## Results

### Breeding performance

The first egg-laying of the Azure-winged magpie population occurred in the same period in both 2018 and 2019 (April 22 in 2018, 2019), while the first egg-laying in the last nest occurred a little later in 2019 (May 30 in 2018; June 11 in 2019). The clutch size of the breeding population was 7.3 ± 0.8 (*n* = 11 nests) in 2018 and 6.9 ± 0.8 (*n* = 16 nests) in 2019 on average. The brood size averaged 7.0 ± 1.0 (*n* = 11 nests) in 2018 and 5.8 ± 1.7 (*n* = 15 nests) in 2019. The nest position in the tree (Vertical height from the ground to the nest) was at an average of 238.0 ± 73.0 cm (*n* = 27 nests).

### Nest characteristics and composition

The nest structure of the Azure-winged magpies is easily distinguished into an outer nest and an inner cup by observing the differences between outer structural materials and the inner lining materials (Fig. 1C). The nests collected during the two breeding seasons were measured in detail by dividing the structure (Table 1). The average nest diameter parallel to the long axis was 1.3 ± 0.1 times longer (*n* = 27 nests) than the nest diameter perpendicular to the long axis. The ratio of cup diameters was 1.2 ± 0.1 (*n* = 27 nests) on average. There was no difference in measurements of the most of variables between two breeding seasons, except the base thickness of the outer (t = 3.269, d.f. = 19.113, *p* = 0.004) and inner cup (t = −2.062, d.f. = 25, *p* = 0.0497) and the mass of cup (t = −2.966, d.f. = 25, *p* = 0.007). There was also no significant difference in the measurement of each variable between breeding successful and failed nests (*p* > 0.05 for all variables).

**Table 1:** Mean ± SD values for structural dimensions of the Azure-winged magpie nests.

	Mean ± SD (*n*)			Comparison of breeding season	
Variables	2018 season	2019 season	Total season	*t*	*P*
Nest diameter parallel to long axis (mm)	349.2 ± 45.0 (11)	345.1 ± 56.7 (16)	346.8 ± 51.3 (27)	−0.276	0.785
Nest diameter perpendicular to long axis (mm)	261.6 ± 37.1 (11)	264.1 ± 26.5 (16)	263.0 ± 30.6 (27)	0.305	0.763
Cup diameter parallel to long axis (mm)	193.6 ± 17.5 (11)	187.8 ± 17.7 (16)	190.2 ± 17.5 (27)	−0.826	0.417
Cup diameter perpendicular to long axis (mm)	157.1 ± 16.2 (11)	158.1 ± 12.1 (16)	157.7 ± 13.6 (27)	0.250	0.805
Nest height (mm)	94.4 ± 6.6 (11)	104.1 ± 15.9 (16)	100.1 ± 13.6 (27)	2.043	0.053
Cup height (mm)	63.6 ± 7.9 (11)	62.8 ± 12.8 (16)	63.2 ± 11.0 (27)	−0.362	0.721
Maximum cup depth (mm)	42.7 ± 5.8 (10)	42.3 ± 10.7 (16)	42.4 ± 9.0 (26)	0.105	0.867
Cup wall thickness (mm)	30.3 ± 5.0 (10)	31.2 ± 6.7 (16)	30.9 ± 6.0 (26)	0.259	0.797
Outer wall thickness (mm)	77.8 ± 23.2 (11)	78.7 ± 30.2 (16)	78.3 ± 27.1 (27)	−0.044	0.965
Cup base thickness (mm)	22.1 ± 5.6 (11)	17.7 ± 4.8 (16)	19.5 ± 5.5 (27)	−2.062	0.0497
Outer base thickness (mm)	31.0 ± 3.4 (11)	44.1 ± 15.7 (16)	38.8 ± 13.8 (27)	3.269	0.004
Nest mass (g)	277.9 ± 79.0 (11)	318.6 ± 94.6 (16)	302.0 ± 89.3 (27)	1.150	0.261
Outer mass (g)	233.5 ± 63.7 (11)	292.6 ± 56.7 (16)	268.5 ± 85.4 (27)	1.791	0.086
Cup mass (g)	44.4 ± 20.7 (11)	25.8 ± 10.4 (16)	33.4 ± 17.7 (27)	−2.966	0.007
Cup volume (cm^3^)	306.8 ± 62.4 (10)	292.6 ± 75.6 (16)	298.1 ± 67.0 (26)	0.143	0.912

**Figure 2:**
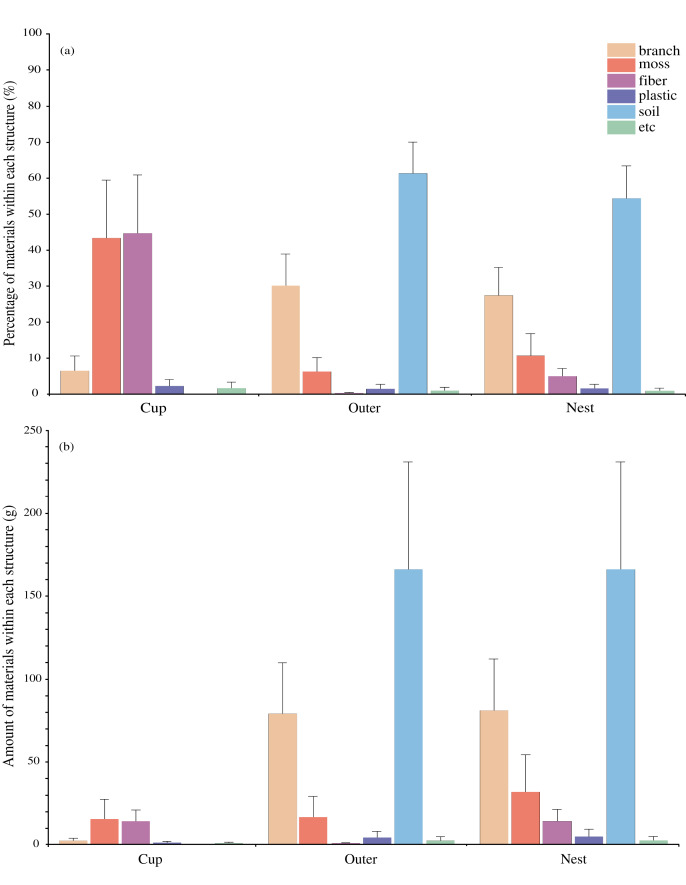
Within-nest variation in the composition of the 27 Azure-winged magpie nests in 2018 and 2019 breeding seasons. (A) The percentage of the different components and (B) the amount of materials used. Values are means +1 standard deviation.

We have analyzed the type and amount of the constituent materials of the 27 Azure-winged magpie nests. Among them, soil represented the largest proportion with 54.7 ± 9% (167.7 ± 65.0g) of the total nest mass, branches constituted 27.5 ± 7.8% (80.8 ± 31.5g), moss 10.6 ± 6.1% (32.1 ± 22.6g), fiber 4.8 ± 2.2% (14.1 ± 7.1 g), plastic 1.5 ± 1.2% (4.8 ± 4.6 g), and others (a small amount of materials such as seeds, broken eggshells, cocoons, and a dead caterpillar) were 0.8 ± 0.8% (2.3 ± 2.4 g) in order (Fig. 2A). The type and mass of the materials in the Azure-winged magpie nest differed between the outer nest and the inner cup (Fig. 2B). In the outer, it was composed of soil (167.7 ± 65.0 g), branch (78.8 ± 31.2 g), moss (16.7 ± 12.7 g), plastic (4.8 ± 4.6 g), fiber (0.4 ± 0.4 g), and others (1.9 ± 2.3 g). The inner cup was composed of moss (15.4 ± 12.1 g), fiber (13.7 ± 7.1 g), branch (2.0 ± 1.3 g), plastic (0.8 ± 0.9 g), and others (0.4 ± 0.6 g). There was no difference in the amount of each material between two breeding seasons, except three materials such as branch in the outer and, moss and fiber in the inner cup (t = 2.265, d.f. = 25, *p* = 0.032 for the branch; t = −3.424, d.f. = 25, *p* = 0.002 for moss; t = −2.270, d.f. = 25, *p* = 0.032 for fiber).

### Relation to ecological factors

**Figure 3:**
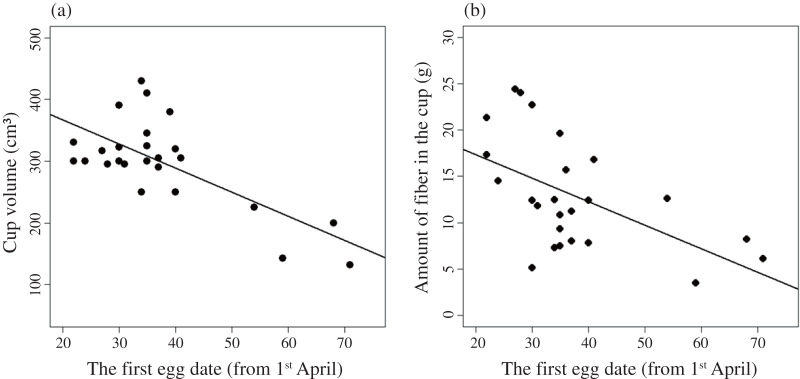
The relationship between the timing of breeding and **(A) cup volume, and (B) the amount of fiber in inner cups of the 25 Azure-winged magpie nests**.

In analyses the relationship between nest characteristics and ecological factors, our results showed that most ecological factors were not significantly related to the size, mass and cup volume of the Azure-winged magpie nest, except of the timing of breeding (χ^2^ = 24.838, d.f. = 1, *p* < 0.001; Table 2). The volume of inner cup decreased with the timing of breeding (t = −4.984, *p* < 0.001; Fig. 3A and Appendix 2). In the case of materials in the cup, the amount of moss did not change as the breeding season progressed (t = −0.127, *p* = 0.900), but the amount of fiber decreased (t = −3.089, *p* = 0.005; Fig. 3B). In the analysis for the relationship between the breeding output and nest characteristics, our results showed that breeding success was not related to the size, mass and cup volume of the breeding nest (Table 3).

**Table 2:** Summary of general linear models analyzing the size, mass and cup volume of 25 Azure-winged magpie’s nest in relation to ecologically relevant factors.

	Nest size				Nest mass				Cup volume*			
Variables	Estimate ± SE	*d.f*.	*χ* ^*2*^	*P*	Estimate ± SE	*d.f*.	*χ* ^*2*^	*P*	Estimate±SE	*d.f*.	*χ* ^*2*^	*P*
Year	–	1	0.199	0.661	–	1	1.700	0.208	–	1	0.005	0.945
Timing of breeding	–1.971 ± 1.647	1	1.431	0.246	–0.600 ± 1.684	1	0.127	0.726	–0.134 ± 0.027	1	24.838	<0.001
Site	–	1	1.271	0.274	–	1	0.095	0.761	–	1	0.452	0.510
Nest position	0.077 ± 0.261	1	0.087	0.772	–0.290 ± 0.267	1	0.177	0.292	0.008 ± 0.004	1	3.773	0.067
No. of neighbors	−45.210 ± 48.735	1	0.861	0.365	–21.278 ± 49.811	1	0.183	0.674	–0.359 ± 0.798	1	0.203	0.658

**Note:**

* The value was transformed using the square root.

**Table 3:** Results of a generalized linear model analyzing breeding success in relation to the size, mass, and cup volume of the 25 Azure-winged magpie nests.

Parameters	Estimate ± SE	Z value	*P*
(Intercept)	0.038 ± 2.810	0.014	0.989
Nest size	–0.004 ± 0.007	–0.520	0.603
Nest mass	0.005 ± 0.007	0.718	0.473
Cup volume	–0.002 ± 0.007	–0.325	0.745

## Discussion

The nests of the Azure-winged magpie might be divided into two parts–the outer nest and the inner cup–and it is easy to visually distinguish these separate parts since the materials used in their construction are different (Fig. 1C). This structure is similar to the case of the Bullfinch (*Pyrrhula pyrrhula*) nest (Bocheński & Oleś, 1981; Biddle, Goodman & Deeming, 2017). In the Azure-winged magpie nest, the outer nest is mainly constructed with branches and soil and constitutes the overall shape of the nest. The base of the outer nest is composed of soil as well as some branches and moss, and the soil appears to act as an adhesive to hold the lowest part of the nest to the tree trunk. The wall of the outer nest predominately consists of branches and provides a stable shape surrounding a direct breeding space like the inner cup. The cup located inside the outer nest is made of more flexible and softer materials compared to the materials constituting the outer nest (Fig. 2). The primary function of the cup in most nests is to insulate the inside of the nest and to keep the nestlings safe from coarser materials (Lovette & Fitzpatrick, 2016). Since the cup is a structure for incubating eggs and raising nestlings, materials that are elastic and have insulation properties such as fiber and moss are used instead of more rigid and coarser materials such as branches. What is unusual about Azure-winged magpie nests is that plastic was used as a constituent material (Fig. 2), and most pieces of plastic pieces looked like hard branches or strings (they turned out to be mainly cable ties or plastic ropes). Hard pieces are mainly used between branches in the outer nest and appear to act as branches. Also, the soft pieces of plastic like strings or fibers inside the cup. This diversity of materials shows that birds distinguish between different types of materials and their relevant characteristics when selecting the materials used to build their nests (*e.g*., Bailey et al., 2014; Biddle, Deeming & Goodman, 2015; Biddle, Goodman & Deeming, 2017).

In our results, most ecological factors were not significantly related to the size, mass and cup volume of the Azure-winged magpie nest (Table 2). First, it is known that the positioning of the nest can affect overall size. This is caused by the differences in predation pressures relative to the position of the nest in the tree (Lima, 2009; Tabib et al., 2016). However, the previous study showed that the vertical location of nest in the tree did not affect the reproductive success of Azure-winged magpies in our study population (Kim et al., 2020). Therefore, it can be concluded that the nest position was not related to the nest size in our analysis. Second, it was anticipated that the nest size in the high nest density area would be small due to the influence of resource competition. However, the number of neighboring nests had no effect on the size of the Azure-winged magpie nests. Considering the life-history features of Azure-winged magpies such as colonial breeding and cooperative breeding, it appears that the number of neighboring nests does not relate to the overall size of the nest.

In our study, we found that both the cup volume and the amount of fiber decreased with the timing of breeding (Fig. 3). The changes in the cup volume seem to be associated with the changes in the amount of fibers constituting the cup. The change in the volume and the amount of fiber in the cup in relation to the timing of breeding may be related to the efficiency of maintaining the nest insulations. In general, the nest characteristics such as the nest size and materials can affect the function of maintaining the appropriate temperatures required for incubation and brooding, *i.e*. insulation properties (Rohwer & Law, 2010; McClintock, Hepp & Kennamer, 2014). In particular, it is necessary to keep temperature changes minimal in order for the embryos to develop and for the nestlings to hatch since exposure to high or low temperatures can be fatal to the embryos (DuRant et al., 2013). As nest size increases, the insulation effect of the nest can also increase. Consequently, breeding in a relatively large nest during a hot period may result in ineffective cooling of eggs or nestlings (Møller, 1982). Moreover, with open nests, it is relatively difficult to maintain an appropriate temperature compared to cavity nests, which are relatively less exposed (Windsor, Fegely & Ardia, 2013). In our study, the breeding of the Azure-winged magpie took place from spring to early summer, and the temperature of the study site increased over time. In the end, breeders that attempted to breed later chose to reduce the insulation effect by controlling the amount of fiber in the cup because they needed to maintain an appropriate temperature for their eggs and nestlings. These results are similar to the results from the study on Prairie Warblers (*Setophaga discolor*), which showed that the nest size was affected by thermal properties (Akresh, Ardia & King, 2017).

In the study population, the main cause of breeding failure is predation (Kim et al., 2020). The selection of a nesting site and the design of the nest can be contributing factors to successful breeding, since the larger the nest size, the more it can be exposed to predators (Mainwaring et al., 2014). Indeed, several studies report that the predation rate increased with the nest size (Snow, 1978; Slagsvold, 1989; Biancucci & Martin, 2010). In our results, however, the nest size and mass were not related to the breeding success (Table 3). According to a previous study conducted on the same breeding population of Azure-winged magpies (Kim et al., 2020), it was observed that predation was more prevalent in nests located outside the breeding site. Therefore, taking into account these results, it is considered that the degree of predation was based largely on the location of the nests within the colony.

## Conclusion

In this study, we presented that the nests of Azure-winged magpie exhibit structural characteristics that are divided into an outer nest and an inner cup, and that materials suitable for each part of the nest were used when constructing the nest. We also found that the size and mass of their nests were not related to the ecologically relevant factors. However, the volume of the inner cup decreased as the breeding season progressed. Even though the sample size was small in our study, we can conclude that the Azure-winged magpie is able to control the amount of fiber used in the construction of the inner cup in response to ambient temperature. Therefore, further research is needed to shed light on the mechanisms of thermal control in nests relative to changes in the surrounding environment during the breeding season.

## Supplemental Information

10.7717/peerj.13637/supp-1 Supplemental Information 1 Mean temperature during the breeding season.

10.7717/peerj.13637/supp-2 Supplemental Information 2 Results of a linear model analyzing the cup volume of 25 Azure-winged Magpie’s nests in relation to ecologically relevant factors.

10.7717/peerj.13637/supp-3 Supplemental Information 3 Measurement dataset.

## Additional Information and Declarations

### Data Availability

The following information was supplied regarding data availability: The raw measurements are available in the Supplemental Files.
