# Supplementary figures and images for: Nest characteristics and composition of the colonial nesting Azure-winged magpie Cyanopica cyanus in South Korea
Source: PeerJ. 2022 Jun 29;10:e13637. doi: 10.7717/peerj.13637 (PMC9250309; doi:10.7717/peerj.13637)

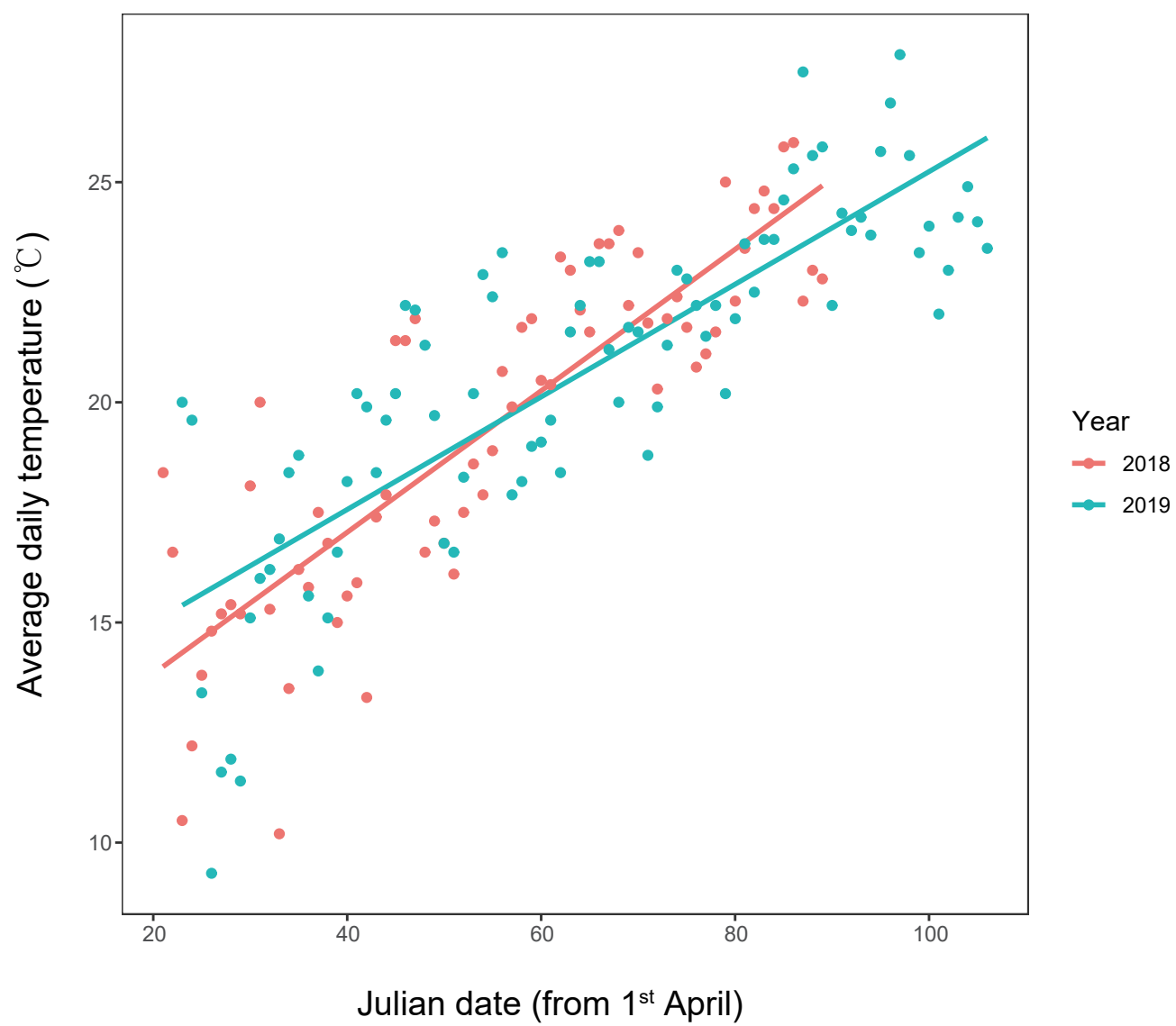

Supplement: Supplemental Information 1 [file peerj-10-13637-s001.pdf]
