# Supplementary material for: Nest characteristics and composition of the colonial nesting Azure-winged magpie Cyanopica cyanus in South Korea
Source: PeerJ. 2022 Jun 29;10:e13637. doi: 10.7717/peerj.13637 (PMC9250309; doi:10.7717/peerj.13637)
Supplement: Supplemental Information 2 [file peerj-10-13637-s002.docx]

Appendix 1. Results of a linear model analyzing the cup volume of 25 Azure-winged Magpie’s nests in relation to ecologically relevant factors

|  | Estimate | Std. Error | t value | Pr (>\|t\|) |
| --- | --- | --- | --- | --- |
| (Intercept) | 20.409127 | 1.249364 | 9.169 | 2.09e-08 *** |
| Year (2019) | 0.043418 | 0.618999 | 0.070 | 0.9448 |
| Timing of breeding | –0.134404 | 0.026968 | –4.984 | 8.24e-05 *** |
| Site (S2) | –0.438823 | 0.652751 | –0.672 | 0.5095 |
| Nest position | 0.008306 | 0.004276 | 1.942 | 0.0671 |
| No. of neighbors | –0.359382 | 0.797843 | –0.450 | 0.6575 |
| Multiple R-squared | 0.6351 | |  |  |
| Adjusted R-squared | 0.539 | |  |  |
| F-statistic | 6.613 on 5 and 19 DF | |  |  |
| P value | 0.001008 | |  |  |
